# Study on Morphology, Age and Growth of River *Perca fluviatilis* in Kalasuke Reservoir, Xinjiang, China

**DOI:** 10.3390/ani15172469

**Published:** 2025-08-22

**Authors:** Wenjun Li, Guanping Xing, Zhengwei Wang, Shuangshuang Liang, Huale Lu, Yunhong Tan, Jie Wei, Zhulan Nie

**Affiliations:** 1 College of Life Science and Technology, Tarim University, Alar 843300, China; 10757231128@stumail.taru.edu.cn (W.L.); 10757231125@stumail.taru.edu.cn (G.X.); 10757232145@stumail.taru.edu.cn (Z.W.); 4301223315@stumail.taru.edu.cn (S.L.); 10757241102@stumail.taru.edu.cn (H.L.); 2201223121@stumail.taru.edu.cn (Y.T.); 2 Xinjiang Production & Construction Corps Key Laboratory of Protection and Utilization of Biological Resources in Tarim Basin, Alar 843300, China

**Keywords:** Kalasuke Reservoir, *Perca fluviatilis*, age structure, growth feature, individual morphology

## Abstract

In late August and mid-November 2024, and late February and mid-May 2025, four surveys were conducted in the Kalasuke Reservoir section of the Irtysh River, resulting in the collection of 296 samples of *P. fluviatilis*. Sampling tools included drift gillnets with a mesh size of 5 cm and an outer mesh size of 10 cm, bottom cages with a mesh size of 1 cm, and fishing rods (4.5 m and 5.4 m). The age structure and growth characteristics of *P. fluviatilis* in the reservoir were analyzed. Results showed that the body length of the sampled fish ranged from 100.53 to 305.30 mm, with the dominant length group being 100.53–150.00 mm, accounting for 90.09% of the total. Body mass ranged from 24.20 to 490.20 g, with the dominant mass group below 66.5 g, accounting for 89.86%. The age composition of the population consisted of age classes 1–5, with ages 1–2 years old being dominant, accounting for 96.2% of the total samples. Among these, 1-year-old individuals were the most abundant, accounting for 78.3%, while older fish were relatively scarce. The relationship between body length (*L_t_*) and body mass (*W_t_*) was modeled as *W_t_* = 4.298 × 10^−5^ *L_t_*^2.85^ (*R*^2^ = 0.998, *n* = 296). The von Bertalanffy growth equations were *L_t_* = 652.866 [1 − *e*^−^^0.108^(*^t^*^+0.778^)] and *W_t_* = 4990.21 [1 − *e*^−^^0.108^(*^t^*^+0.778^)]^2.85^, with a growth coefficient *K* = 0.108. The inflection point of growth was determined to be 1.9 years by fitting growth rate and acceleration equations. The *b* < 3 indicates allometric growth, where body length increases faster than body mass, suggesting that *P. fluviatilis* prioritizes elongating its body to enhance swimming ability and expand its range, while accumulating muscle and fat at a slower pace. Principal component analysis (PCA) revealed that the cumulative contribution rate of the first three principal components was 55.45%, reflecting the morphological characteristics of the species. The accuracy of discriminant analysis for sex determination based on external morphology was 67.20%, indicating limited reliability in gender identification using only morphological traits.

## Simple Summary

In 2024 and 2025, 296 river *Perca fluviatilis* samples were collected four times from Kalasuke Reservoir in Xinjiang to study their morphology, age, and growth. *Perca fluviatilis* has a long elliptical body with typical characteristics. Principal component analysis showed that the cumulative contribution rate of the first three principal components of their morphology was 55.45%, and the accuracy of sex discrimination by external morphology was 67.20%. Most fish were 1 year old. The length-weight relationship indicated allometric growth. The von Bertalanffy equation described their growth with specific parameters. Growth rate decreased with age and varied by sex. These results provide basic data for fishery management, highlighting the fish’s adaptability and the need to consider multiple factors in management, contributing to population assessment, resource protection, and rational fishing.

## 1. Introduction

The Kalasuke Reservoir is located in Fuyun County, Altay Prefecture, Xinjiang Uygur Autonomous Region. It is a large-scale water conservancy project in the middle reaches of the Irtysh River. The reservoir has a vast water area, with a total storage capacity of 2.42 billion cubic meters and a regulating storage capacity of 1.92 billion cubic meters. The maximum dam height is 121.50 m, the normal storage water level is 739 m, and the dead water level is 680 m. The terrain around the reservoir is complex and diverse, with various landforms such as mountains, hills, and plains interwoven, creating a rich and diverse habitat environment for the *Perca fluviatilis* [1]. Its water source mainly comes from the Irtysh River, and the water quality is excellent. The dissolved oxygen content remains above 5 mg/L throughout the year, and the pH value is stable between 7.5 and 8.5, which is extremely suitable for the survival and reproduction of a variety of aquatic organisms. The *P. fluviatilis* is one of the important fish populations in this area. In recent years, with the vigorous development of the fishery economy in the surrounding areas, the fishing intensity of the *P. fluviatilis* has been increasing day by day, which poses a potential threat to its population size and structure. Therefore, it has become an urgent task to deeply explore the age and growth laws of the *P. fluviatilis* [2].

Abroad, the research and farming of the *P. fluviatilis* have a long history. Europe, as the main distribution area of the *P. fluviatilis*, has conducted in-depth and comprehensive research on its biological characteristics, covering many aspects such as growth laws, reproductive habits, and ecological niches [3]. In the study of growth laws, it has been clarified that the *P. fluviatilis* grows relatively fast in the 6–7 years after sexual maturity. In terms of reproductive habits, it has been found that its spawning period is relatively early. After the lake thaws in late April, when the water temperature reaches 6–8 °C, the *P. fluviatilis* begins to spawn [4]. From an ecological perspective, the *P. fluviatilis* has strong ecological invasiveness, a strong reproductive capacity, and its population can grow rapidly. However, when the water environment deteriorates or the fishing intensity is too high, the *P. fluviatilis* resources will decline. Fortunately, this species has a relatively strong recovery ability. In the field of aquaculture, in the past 25 years, *P. fluviatilis* farming has gradually become an important direction for the diversified development of inland aquaculture in Europe. Its domestication process has gone through several stages [5,6].

In China, the *P. fluviatilis* is mainly distributed in the Irtysh River, Ulungur Lake, and other basins in Xinjiang [7,8]. Scientific researchers have carried out a large number of studies on the *P. fluviatilis* in the Irtysh River. For example, it has been determined that its maximum lifespan can reach 22.2 years. In the research of genetic diversity, the Heilongjiang Fisheries Research Institute collected samples from Ulungur Lake, Jili Lake, Ulungur River, and Kara Irtysh River, respectively [9]. By using the analysis method of screening microsatellite markers based on genomic data, it was found that the populations in Ulungur Lake, Jili Lake, and Ulungur River show a high degree of polymorphism, while the population in the Kara Irtysh River is moderately polymorphic. The research also shows that there is a moderate degree of genetic differentiation among the *P. fluviatilis* populations in Xinjiang. It is speculated that the construction of water conservancy projects has hindered the free movement of the *P. fluviatilis*, which is one of the reasons for the differences [10]. This paper takes the *P. fluviatilis* samples collected from the Kalasuke Reservoir as the research object, and uses the sagittal otoliths as the main material for age determination to conduct an in-depth identification of the age and growth characteristics of the *P. fluviatilis*. The aim is to provide crucial basic data support for a comprehensive understanding of the fish resources in the Irtysh River Basin.

## 2. Materials and Methods

### 2.1. Sample Collection and Processing

**Figure 1:**
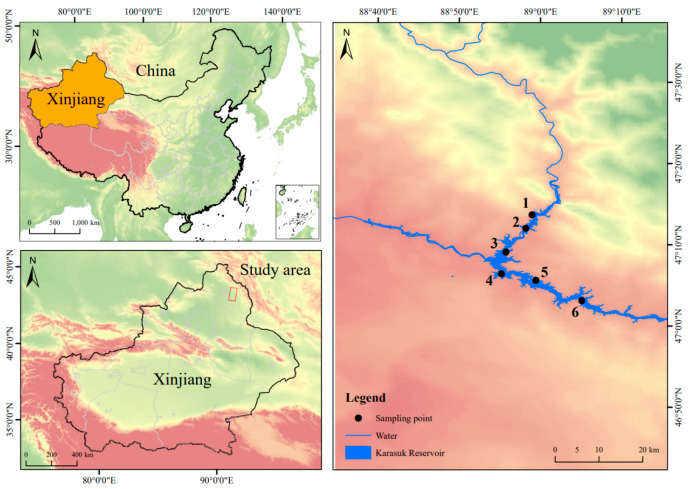
Distribution map of sampling points in the Kalasuke Section of the Irtysh River. Points 1 to 6 represent six different sampling sites, and each sampling site is at least 2 km apart from the others.

From late August 2024, mid-November 2024, and late February 2025 to mid-May 2025, a total of 296 samples were collected from the Kalasuke Reservoir (88°50′0″ E–89°10′0″ E, 47°00′0″ N–47°10′0″ N) section of the Irtysh River in Xinjiang. Each survey period includes 6 sampling sites, each of which has been marked on the map, and the distance between each sampling site is at least 2 km (Figure 1). Seine nets (with an inner mesh size of 5 cm and an outer mesh size of 10 cm), bottom cages (with a mesh size of 1 cm), and fishing rods (4.5 m and 5.4 m) were employed. The samples were subjected to routine biological measurements and sex identification in their fresh state. Subsequently, they were anesthetized with MS-222 (35 mg/L) prior to dissection. The otoliths were removed and placed in 0.2 mL centrifuge tubes, which were then filled with 95% ethanol for immersion. After visually assessing the gonadal development stage, the gonads were weighed and fixed in Bouin’s solution. After determining the food fullness of the intestines, the intestines were soaked in a 10% formalin solution (produced by Tianjin Fuchen Chemical Reagent Co., Ltd., Tianjin, China), and then intraperitoneally injected. Finally, they were transported back to the laboratory for further processing. The length and weight measurements were accurate to 0.01 mm and 0.01 g, respectively. All data were collated, analyzed, and plotted using Excel 2016, SPSS 27.0, and Origin 2022 [11,12,13].

### 2.2. Biological Determination

#### 2.2.1. Traditional Morphological Measurement

**Figure 2:**
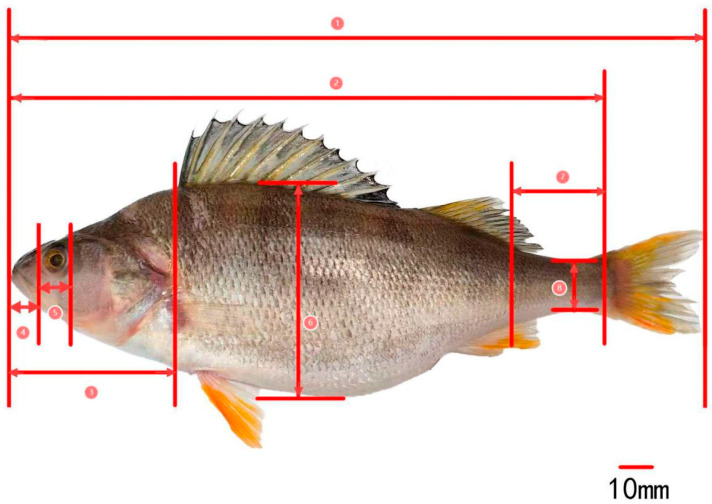
Morphological measurement of *Perca fluviatilis*—1: total length; 2: body length; 3: head length; 4: snout length; 5: eye diameter; 6: body depth; 7: caudal peduncle length; 8: caudal peduncle height.

The measurement indicators for the traditional morphology of *P. fluviatilis* (Figure 2) mainly include total length, (TL); body length, (BL); body depth, (BD); body width, (BW); head length, (HL); snout length, (SL); eye diameter, (ED); eye spacing, (ES); caudal peduncle length, (CPL); caudal peduncle height, (CPH); mouth cleft wide, (MCW); and mouth cleft high, (MCH) [14,15,16].

#### 2.2.2. Truss Morphometry Measurement

**Figure 3:**
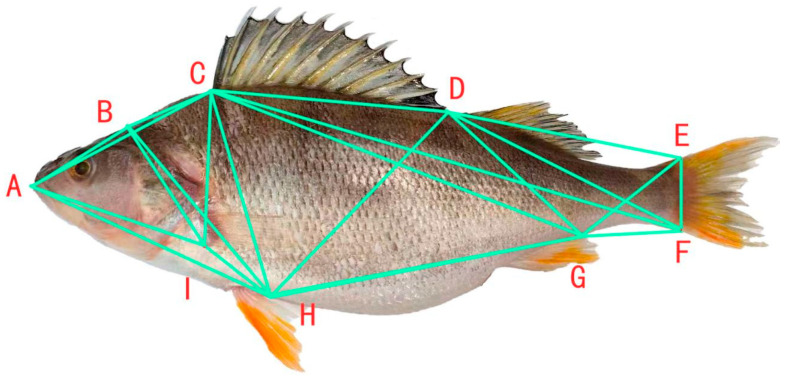
Truss morphometry measurement of *Perca fluviatilis*. Note: A. Tip of the snout; B. back of the occiput; C. origin of the dorsal fin; D. end of the dorsal fin base; E. origin of the dorsal part of the caudal fin; F. origin of the ventral part of the caudal fin; G. end of the anal fin base; H. base of the pelvic fin; I. origin of pectoral fin.

For the framework morphology measurement (Figure 3), coordinate points were selected following the method of Sifa Li et al. [17]. The coordinate points were A, B, C, D, E, F, G, H, and I, respectively. By connecting these representative coordinate points, a total of 24 framework distances were set (Figure 2 and Figure 3), namely A-B, B-C, C-D, D-E, E-F, F-H, H-I, A-I, A-C, C-I, C-H, C-F, D-I, D-H, E-H, D-F, and H-G. Among them, A-B is the distance from the tip of the snout to the back of the occiput, B-C is the distance from the back of the occiput to the origin of the dorsal fin, and so on [18].

### 2.3. Sex Determination

The morphological characteristics of the *P. fluviatilis* ovary at different developmental stages as observed with the naked eye are as follows: Immature Stage: Stage I: The ovary is tightly attached to the coelomic membrane on both sides of the swim bladder, appearing as a transparent thread-like structure. It is difficult to distinguish between males and females with the naked eye, no oocytes are visible, and the surface blood vessels are absent or extremely fine. The ovary has not yet begun to develop at this stage. Stage II: The ovary is mostly flat and ribbon-shaped, with numerous blood vessels distributed on it, which can be distinguished from the testis, but oocytes are still not visible to the naked eye. At this stage, the ovary begins to develop, with increased volume and weight.

Mature Stage: Stage III: The volume of the ovary increases due to the growth of oocytes, and yolk-accumulating oocytes can be clearly seen with the naked eye. However, the oocytes are not large or round enough and cannot be detached from the ovarian folds [19,20]. The ovary significantly enlarges, presenting a vivid yellowish-red color, with fine blood vessels and a tight, elastic texture when pressed. At this point, the eggs begin to accumulate yolk. Stage IV: This is the mature stage, where the volume and weight of the ovary reach their maximum by the end of this stage. It appears beige, almost filling the entire abdominal cavity, with thickened and prominent blood vessels, an elastic texture, and adherent oocytes. Histological observation shows that the oocytes are filled with yolk granules and oil droplets or globules. The ovary is heaviest at this stage, as the *P. fluviatilis* prepares for spawning.

Spawning Stage: Stage V: The ovary is fully mature, with a darkened color, slightly dim orange-red, transparent oocytes, a thin and transparent ovarian membrane, thick and prominently raised blood vessels, and distinct, separate eggs. The texture has poor elasticity, and slight pressure causes the eggs to flow out. At this stage, yolk granules in the oocytes fuse and enlarge, and oil droplets coalesce into oil globules, indicating the stage of impending or ongoing spawning.

Post-spawning Stage: Stage VI: The ovary after spawning appears as a collapsed sac, with congested surface blood vessels. In batch-spawning *P. fluviatilis*, the ovary reverts to Stage II after a short recovery period; in individuals with batch spawning, undeveloped oocytes remain in the ovary, which resumes development and spawns again after a period, with the ovary reverting to Stage IV after recovery.

The morphological characteristics of the *P. fluviatilis* testis at different developmental stages as observed with the naked eye are as follows:

Immature Stage: Stage I: The testis is tightly attached to the coelomic membrane on both sides of the swim bladder, appearing as a transparent thread-like structure. It is difficult to distinguish between males and females with the naked eye, and the testis has not yet developed, with almost no obvious testicular structure visible. Stage II: The testis is ribbon-shaped, semi-transparent or opaque, with inconspicuous blood vessels, mostly grayish-white or brownish-gray in color. Males and females can be preliminarily distinguished, but the testis is small and occupies only a small portion of the abdominal cavity. The testis begins to develop at this stage, with gradual cell proliferation.

Mature Stage: Stage III: The testis increases in volume, appearing as a round rod, firm in texture, smooth surface without wrinkles, with capillary blood vessels and a pale pink color. Squeezing the abdomen or cutting the testis does not produce semen. The testis further develops at this stage, with internal cells beginning to undergo meiosis and other preparatory processes. Stage IV: The testis is milky white, with more prominent surface wrinkles and obvious blood vessel distribution. By the end of Stage IV, a small amount of semen flows out when the testis is pricked or the fish’s abdomen is gently pressed, and the cross-sectional edge of the testis is slightly rounded. This indicates that sperm within the testis are gradually maturing. Stage V: The seminiferous tubules are filled with sperm, and the testis reaches its maximum volume, appearing milky white. Lifting the head of the broodstock or gently pressing the abdomen causes a large amount of thick, milky semen to flow out from the genital opening. This is the stage when the testis is fully mature and ready for or in the process of ejaculation.

Post-ejaculation Stage: Stage VI: The testis significantly decreases in volume after ejaculation, becoming a thin ribbon-like structure and turning light red in color. The testis is in an atrophic and relaxed state, with possible residual semen inside. This stage marks the beginning of the preparation for the next developmental cycle, typically regressing to Stage III for redevelopment [21,22].

The ovarian development of *Perca fluviatilis* exhibits distinct staged characteristics: Stage I is the immature stage, with the ovary appearing as a thin thread-like structure, transparent and without obvious ova. Stage II sees the ovary gradually enlarging into a slender ribbon shape, turning pale yellow, with ova starting to form but remaining indistinct. Stage III is marked by significant volume expansion, occupying one-third to one-half of the abdominal cavity, where ova become visible with uneven sizes and initial yolk deposition. Stage IV features a plump ovary containing uniformly sized ova filled with yolk, occupying more than two-thirds of the abdominal cavity. Stage V is the mature stage, with free ova that can be extruded by gently pressing the abdomen and are capable of fertilization. Stage VI is the post-spawning recovery stage, during which the ovary shrinks, becomes soft in texture, retains degenerated ova, and gradually returns to the state of Stage II, awaiting the next developmental cycle. This process, regulated by environmental factors, constitutes the core link of its reproductive cycle [23].

### 2.4. Age Determination Using Otoliths

**Figure 4:**
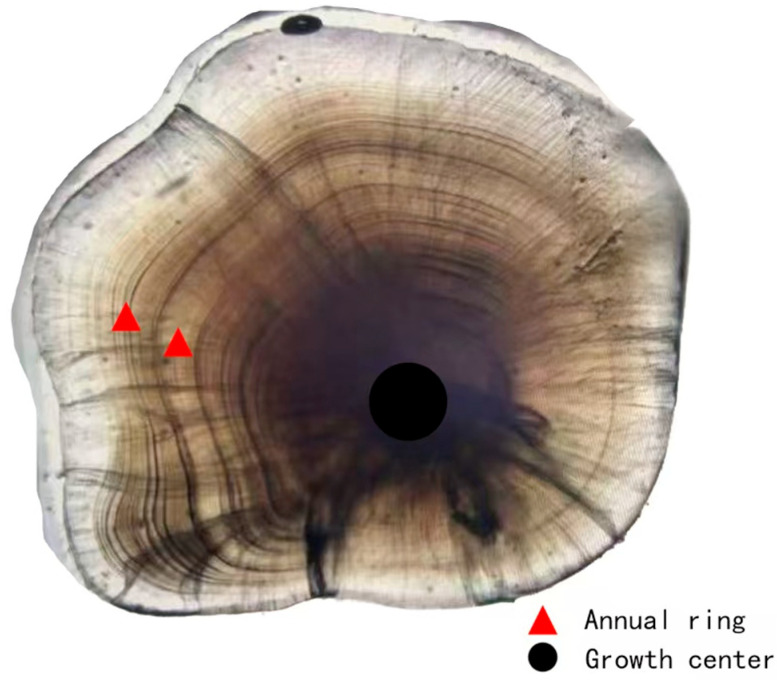
Images of *Perca fluviatilis* otoliths—Black circles represent annual, and red triangles represent growth.

The age of *P. fluviatilis* was determined using sagittal otoliths (Figure 4) [24]. A pair of sagittal otoliths was removed from each *P. fluviatilis*, embedded in nail polish, and left to set for 12 h. The otoliths were then polished in a circular motion with 5000-grit sandpaper, with regular microscopic observation during the process [25]. Once the polishing reached the central region of the otolith, the otolith was flipped, and the procedure was repeated on the other side. When clear annual rings became visible, they were observed, counted under a microscope, photographed, and preserved. The age was determined through blind inspection by 2–3 observers. Microscopic observation revealed that each otolith annulus consisted of a transparent zone and a dark zone. In most otoliths, the marginal area had formed the growth dark zone of the next annulus; therefore, these otoliths were assigned to the next age class and recorded as integers. For example, individuals with a growth stage of 1+ were recorded as age 2, those with 2+ were recorded as age 3, and so on [26,27,28].

### 2.5. The Relationship Between Body Length and Body Weight

The relationship between body length and body mass was analyzed using power function regression as $W=aL^{b}$, where W is the body mass, L is the body length, and a and b are constants [15,29].

### 2.6. Growth Equation

The growth characteristics of *P. fluviatilis* were described using the von Bertalanffy growth equation (VBGF) [30].

The standard formula for the von Bertalanffy growth equation is as follows:

$$L_{t}=L_{\infty }\left(1-e^{-k\left(t-t_{0}\right)}\right)$$

Growth rate equation:

$$Wt=W_{\infty }\left(1-e^{-k\left(t-t_{0}\right)}\right)$$

$$dL/dt=L_{\infty }ke^{-k\left(t-t_{0}\right)}$$

$$dW/dt=bW_{\infty }ke^{-k\left(t-t_{0}\right)}{\left(1-e^{-k\left(t-t_{0}\right)}\right)}^{b-1}$$

Growth acceleration equation:

$$d^{2}L/d=-kL_{\infty }e^{-\left(t-t_{0}\right)}$$

$$d^{2}W/dt^{2}=bW_{\infty }k^{2}e^{-k\left(t-t_{0}\right)}{\left(1-e^{-k\left(t-t_{0}\right)}\right)}^{b-2}\left(be^{-k\left(t-t_{0}\right)}-1\right)$$

Growth inflection point age equation:

$$t_{i}=t_{0}+lnb/k$$

Growth characteristic index (φ) equation:

$$\varphi =logk+2logL_{\infty }$$

Note: Lt represents the body length at time t; L∞ represents the theoretical maximum body length; and t represents time (such as age); body length (mm) at age t; body weight (g) at age t; asymptotic body length (mm); asymptotic body weight (g); growth coefficient; assumed theoretical starting age of growth; body length growth rate; body weight growth rate; body length growth acceleration; body weight growth acceleration; and index of the relationship between body length and body weight [14].

## 3. Results

### 3.1. Traditional Morphology

#### 3.1.1. Morphological Description

The *P. fluviatilis* exhibits a streamlined yet distinct body shape. It is laterally compressed and relatively high, with a long elliptical form. The back is arched, the abdomen is straight, and the caudal peduncle is narrow and robust. According to statistics, the total length of fish typically ranges from 119.87 to 355.10 mm. Its body is covered with closely arranged ctenoid scales that are thick and tough. The lateral line is complete and straight, extending along the mid-axis of the body side, accurately outlining the body contour. The body color varies in a gradient according to the habitat. The back is olive-green to dark brown, gradually changing to silver-white toward the abdomen. There are 5–8 distinct dark brown vertical stripes on the body side, which are particularly vivid in juvenile fish, serving as natural camouflage. The pelvic, anal, and caudal fins are bright red-orange. The first dorsal fin has a characteristic black patch on its spinous part, and the posterior-margin forked caudal fin is deeply cleft, with the fork depth approximately one-third of the fin length, providing propulsion for high-speed swimming. The head is compactly structured, slightly pointed at the front. The mouth is terminal, and the oral fissure is oblique, with the lower jaw slightly longer than the upper jaw. Fine villous teeth are distributed on the maxilla, vomer, and palatine bone, adapting to its predatory strategy. The eyes are of moderate size, located laterally and superiorly. The iris is yellow, and the pupil is round, endowing it with acute visual predation ability. The fins have unique morphological features. The dorsal fin is divided into two parts: the anterior spinous part has 9–13 spines, with a striking black patch on the interspinous membrane, and the posterior soft-rayed part has 12–17 soft rays that are flexible. The anal fin has 3 firm spines and 7–10 extended soft rays, with its origin opposite to the soft-rayed part of the dorsal fin. The pelvic fins are thoracic, with red fin rays that can reach the anus. The pectoral fins are broad and short, located inferolaterally, with their tips near the base of the pelvic fins. Sexual dimorphism is prominent. Female individuals are, on average, 10–15% longer in total length than males. Their body cavities are wider to accommodate a large number of eggs (a single female can carry up to 200,000 eggs), and their pectoral and pelvic fin rays are longer, adapting to the movement requirements during the spawning period. Males have a slender body shape, and their fin rays are relatively shorter and stronger, facilitating courtship chasing.

The dorsal fin is divided into two parts: the anterior dorsal fin has spines (8–16 spines), and the posterior dorsal fin has soft rays (12–19 soft rays). The anal fin has 3 spines and 7–10 soft rays.

#### 3.1.2. Measurable Traits

Among the 12 morphometric traits of *P. fluviatilis* (Table 1), body height (19.74–90.13) slightly exceeds body width (10.36–56.02), caudal peduncle length (14.58–76.53) is also longer than caudal peduncle height (5.31–24.12), and mouth width (5.35–24.05) is slightly larger than mouth height (4.60–20.07). Among the standard deviations of the 12 morphometric traits, only those of total length and body length are relatively large, while others are relatively small. This indicates that the perch samples collected in this study exhibit diverse body size characteristics with a wide coverage range.

To reduce the influence of individual size on experimental results, the ratio relationships between morphometric traits of females and males were calculated (Table 2). According to the standard deviations of trait ratios, the variation range of total length/body length was the smallest between female and male individuals, while that of body length/eye diameter was the largest.

#### 3.1.3. Principal Component Analysis (PCA)

After excluding perch individuals of indeterminate sex, a principal component analysis (PCA) was performed on the ratios of 14 measurable morphological traits. The contribution rates of the first three principal components were calculated as follows (Table 3): PC1 (23.29%), PC2 (17.35%), and PC3 (14.81%), with a cumulative contribution rate of 55.45%.

**Figure 5:**
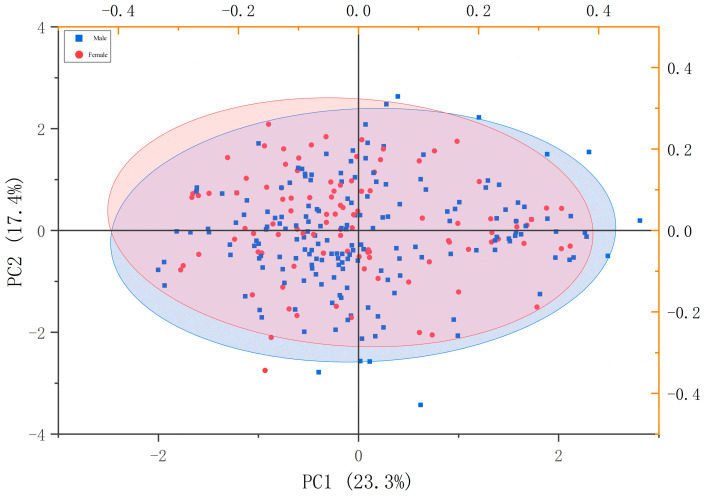
*Perca fluviatilis* measurable trait ratios’ characteristic values in PC1–PC3.

In PC1, the trait ratios playing a dominant role are body length/body width and body length/caudal peduncle length. In PC2, the dominant trait ratios are primarily head length/interorbital distance (appearing twice in the original description, which may be a repetition; consider confirming the trait names). In PC3, the dominant trait ratios are head length/eye diameter and body length/head length. In the constructed two-dimensional scatter plot (Figure 5), there is a significant overlap between male and female individuals of *P. fluviatilis*, indicating that it is difficult to determine the sex of *P. fluviatilis* solely based on external morphological characteristics.

Using discriminant analysis, the ratio parameters of 14 measurable traits of female and male individuals (*n* = 296) of *P. fluviatilis* were analyzed, and a corresponding discriminant function system was established. The specific functional expressions are as follows:

For female *P. fluviatilis*:

$$\begin{array}{l} Y_{1}=733.268X_{1}-628.439X_{2}+1177.675X_{3}+2388.936X_{4}+4518.04X_{5}\\ -484.824X_{6}-397.022X_{7}-462.718X_{8}+15.581X_{9}+1377.754X_{10}\\ +1670.764X_{11}+5.833X_{12}+1569.131X_{13}+61.3X_{14}-10605.85 \end{array}$$

For male *P. fluviatilis*:

$$\begin{array}{l} Y_{2}=732.672X_{1}-629.656X_{2}+1179.612X_{3}+2393.839X_{4}+4517.83X_{5}\\ -486.804X_{6}-397.196X_{7}-461.494X_{8}+15.706X_{9}+1378.494X_{10}\\ +1676.93X_{11}+5.399X_{12}+1565.218X_{13}+60.534X_{14}-10610.267 \end{array}$$

In the formula, X_1_, X_2_, X_3_, X_4_, X_5_, X_6_, X_7_, X_8_, X_9_, X_10_, X_11_, X_12_, X_13_, and X_14_ represent the ratios of total length to body length, body length to body width, body length to body height, body height to body width, body length to head length, body length to caudal peduncle length, body length to eye diameter, body length to interorbital distance, head length to snout length, head length to eye diameter, head length to caudal peduncle length, head length to mouth width, head length to interorbital distance, and caudal peduncle length to caudal peduncle height, respectively. The results show that the comprehensive discriminant accuracy for female and male *P. fluviatilis* is 67.2%.

### 3.2. Truss Morphometry

We performed standardized processing on the linear distances of 9 adjacent coordinate points of the *P. fluviatilis*, with specific results shown in the table (Table 4). Based on the table, the framework structure diagram of the perch was constructed. It can be observed that the perch exhibits a typical laterally compressed fusiform body shape, with a short and slender caudal peduncle. This morphological feature is highly adapted to its ecological habits. Studies have shown that a well-developed caudal peduncle structure can significantly increase the swing amplitude of the fish body and enhance its ability to prey on small aquatic organisms.

### 3.3. Age Estimation

**Figure 6:**
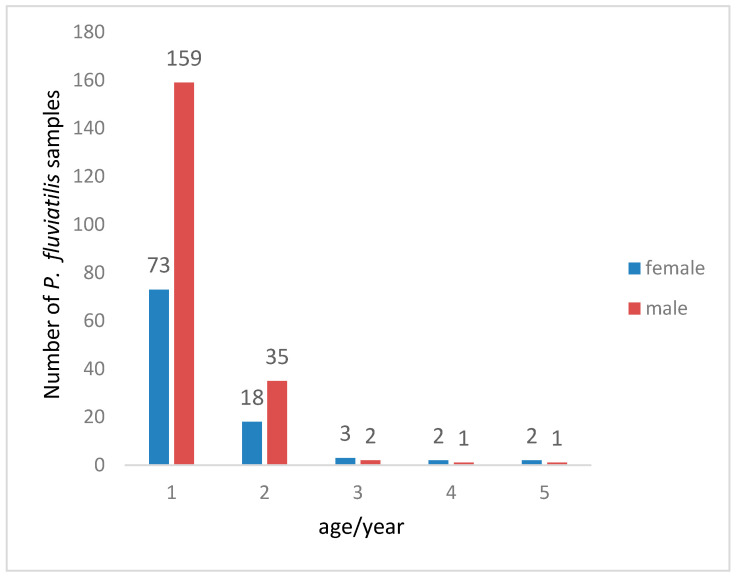
Age composition of *Perca fluviatilis* catches.

A total of 296 *P. fluviatilis* specimens were obtained for age estimation. The population age was composed of 1–5 years old, with the dominant age groups being 1–2 years old, accounting for 96.2% of the total samples. Among them, 1-year-old individuals were the most numerous, accounting for 78.3%, while older fish were fewer. This indicates (Figure 6) that the age structure of the population is relatively simple and shows a trend of younger age.

### 3.4. Population Structure Characteristics

**Figure 7:**
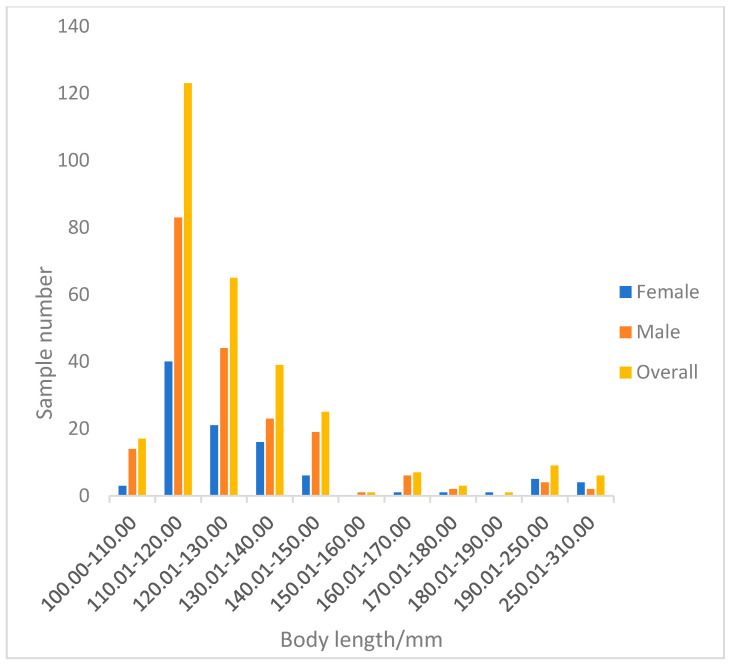
Body length distribution of Perca fluviatilis.

**Figure 8:**
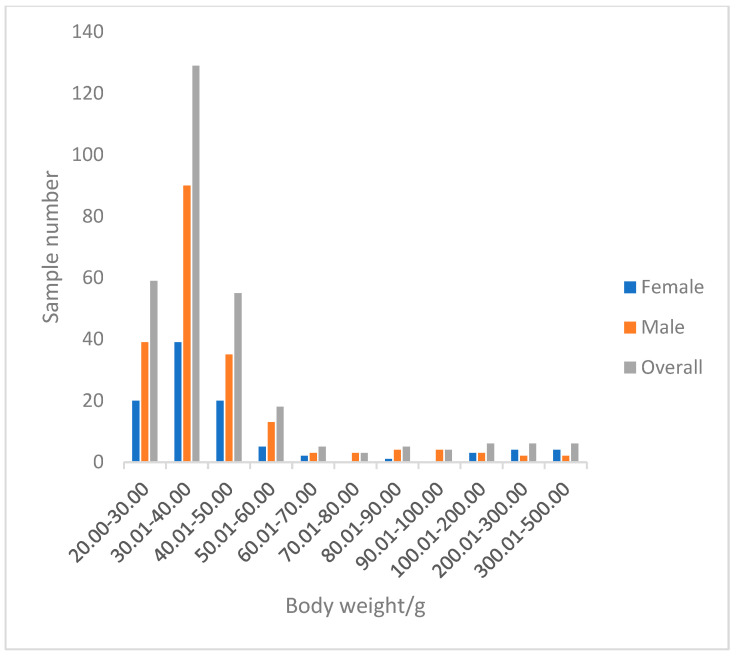
The body weight distribution of *P. fluviatilis*.

A total of 296 perch specimens were measured. The total length of perch ranged from 100.53 to 305.30 mm, with an average of 130.23 ± 29.63 mm. The dominant length was 100.53–150.00 mm, accounting for 90.09% of the total samples (Figure 7). The body mass ranged from 24.20 to 490.20 g, with an average of 52.65 ± 62.53 g. The dominant body mass group was below 66.5 g, accounting for 89.86% of the total samples (Figure 8).

### 3.5. Body Length–Weight Relationship

**Figure 9:**
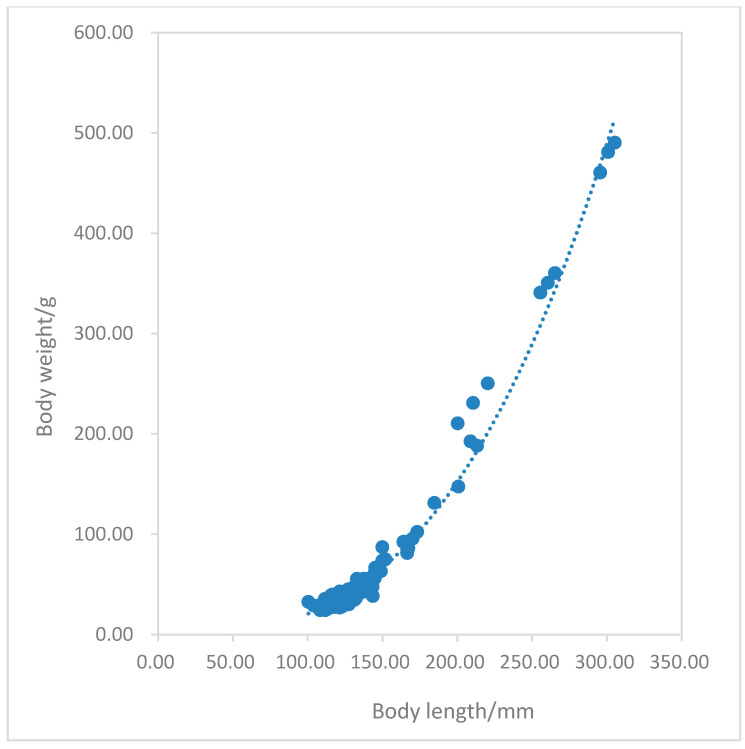
The relationship between the body length and body weight of *Perca fluviatilis*.

The power function was used to carefully fit the correlation between body length and body weight of the overall population and female/male populations of *P. fluviatilis* (Figure 9). Perca fluviatilis exhibited an allometric growth pattern, with body length growth as the main factor (b < 3).

The relationship equation between body length and body weight:

$$W=4.298\times 10^{-5}|L^{2.85}$$

### 3.6. Condition Factor

**Figure 10:**
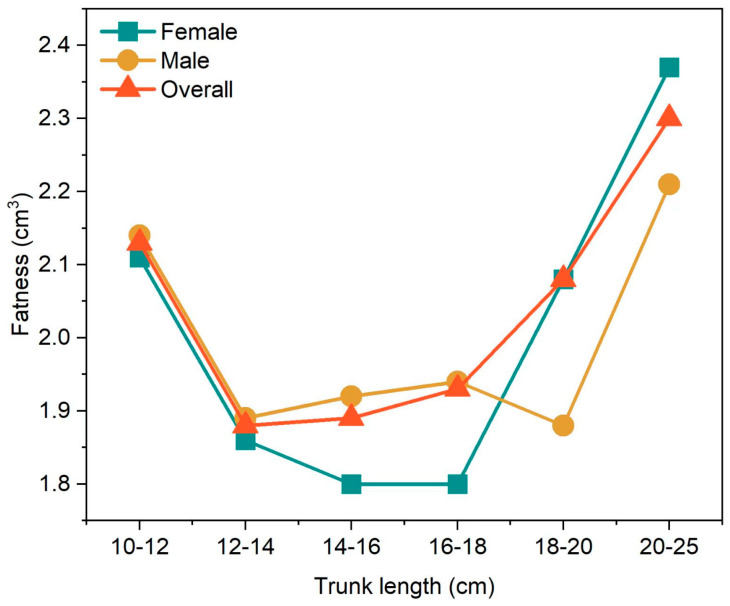
Variation of the condition factor of *Perca fluviatilis* with different body lengths.

To avoid the influence of individual differences on the experimental results, 6 samples with a body length of over 250 mm were excluded, and the condition factor of perch was studied at 2 cm intervals. The results showed that the condition factors of both female and male perch exceeded 1.80 g/cm^3^, and the maximum condition factor in each length interval was 2.37 g/cm^3^ (Figure 10). Overall, the condition factor of female perch was slightly higher than that of male *P. fluviatilis*.

### 3.7. Growth Characteristics

Based on the growth rate and acceleration equations of *P. fluviatilis* in Kalasuke Reservoir derived from the von Bertalanffy growth equation, the study reveals that their growth dynamics exhibit a typical pattern of rapid growth in juveniles and deceleration to stability in adults: The growth rate of body length peaks at 1–2 years old, then shows exponential decay with age. The acceleration is consistently negative, indicating that the growth rate has been in a deceleration phase from the beginning, and the deceleration amplitude slows down after 1.9 years old. The growth rate of body weight is unimodal, reaching the maximum at 2–3 years old. An inflection point occurs at 1.9 years old: the acceleration is positive before this age and turns negative afterward, reflecting that the energy allocation shifts from “body size expansion” to “reproductive reserve” after 1.9 years old. These equations quantify the dynamic changes in key growth stages, providing a scientific basis for fishery management: As the period with the peak growth rate, 2-year-old individuals are the optimal specification for stock enhancement. Individuals over 4 years old have declining growth efficiency and can be set as the main targets for commercial fishing, balancing resource sustainability and economic benefits.

**Figure 11:**
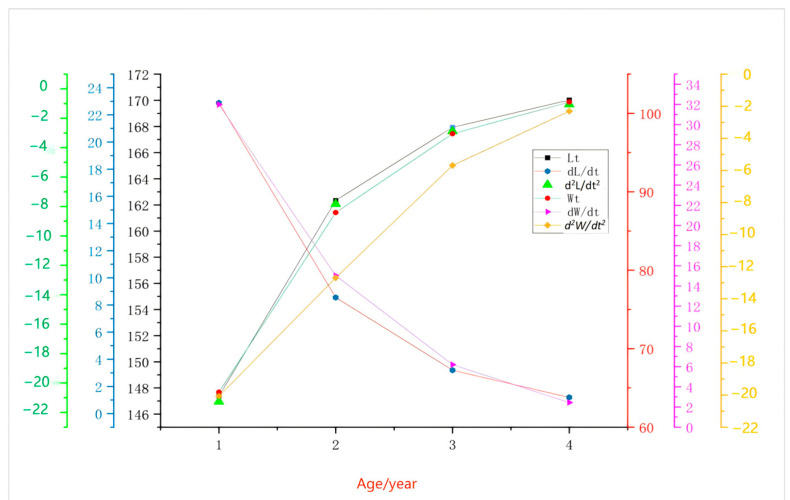
Growth correlation curves of body length and body weight of *Perca fluviatilis*.

By differentiating the von Bertalanffy growth equation, the growth rate and acceleration equations of perch are obtained. The growth-related curves of *P. fluviatilis* are shown in Figure 11.

$$\begin{array}{rr} Lt & =652.866\left[1-e^{-0.108\left(t+0.778\right)}\right]\\ Wt & =4990.21{\left[1-e^{-0.108\left(t+778\right)}\right]}^{2.85} \end{array}$$

The growth rate equation is as follows:

$$\begin{array}{c} dL/dt=70.5e^{-0.108\left(t+0.778\right)}\\ dW/dt=1535.99e^{-0.108\left(t+0.778\right)}{\left[1-e^{-0.108\left(t+0.778\right)}\right]}^{1.85} \end{array}$$

The growth acceleration equation is as follows:

$$d^{2}L/dt^{2}=-12.7e^{-\left(t+0.778\right)}$$

$$d^{2}W/dt^{2}=165.89e^{-0.108\left(t+0.778\right)}{\left(1-e^{-0.108\left(t+0.778\right)}\right)}^{0.85}\left(2.85e^{-0.108\left(t+0.778\right)}-1\right)$$

Growth characteristic index (φ) equation: φ = 4.66.

## 4. Discussion

### Individual Morphological Characteristics

The body of *P. fluviatilis* is long, elliptical, and laterally compressed, a body shape that highly adapts to the environment of the Irtysh River Basin [31]. Its streamlined appearance effectively reduces swimming resistance in water, enabling the perch to shuttle efficiently through turbulent or gentle currents. This is consistent with the principle by which the streamlined body shape of *Opsariichthys bidens* (living in fast-flowing streams) adapts to water currents [32,33]. The perch has a wide mouth with sharp and dense jaw teeth, which endow it with strong predatory capabilities. This oral structure is similar to that of fierce predators such as *Siniperca chuatsi*, demonstrating the perch’s position as a predator in the aquatic ecosystem. Its body scales are tight and thin, providing protection without excessively hindering flexible swimming. The well-developed lateral line system is crucial for the perch to avoid natural enemies, search for food, and locate suitable habitats in the complex and changeable river environment, similar to the function of the lateral line system in many cyprinid fish [2,34]. This study used principal component analysis (PCA) to analyze the proportional relationships of 14 measurable traits in male and female perch. The first three principal components accounted for a cumulative contribution rate of 55.45%, mainly focusing on the head, trunk, and tail [35]. In PC1, the ratios of body length/body width and body length/caudal peduncle length dominated, reflecting that the perch’s body shape is closer to a streamlined type, allowing for faster and more flexible turning [14,30]. This is beneficial for shuttling through complex water environments (such as areas filled with water plants and reefs), reducing swimming resistance, and enabling the perch to swim more efficiently and save energy when pursuing prey or escaping natural enemies [36]. In PC2, the ratio of head length/interorbital distance dominated, indicating that the relative development of the perch’s visual organs is better, which helps improve visual acuity and assists the perch in more accurately locating prey and identifying natural enemies in the complex water environment of the Irtysh River [37]. In PC3, the ratios of head length/eye diameter and body length/head length dominated in the principal component analysis, reflecting that the perch has evolved the ability to hunt in low-light environments of deep water areas and withstand strong currents in this basin. These abilities are key dimensions for the perch to adapt to different predation pressures or habitats. Discriminant analysis has been widely used to distinguish population differences in perch. In this study, discriminant analysis was performed on the ratio parameters of 14 measurable traits of male and female perch, and the sex discrimination accuracy rate was 67.2%. Different from previous studies, the discriminant equation constructed in the morphological and growth analysis of *Micropterus salmoides* (Perciformes, Centrarchidae) included 8 eigenvalues, with an accuracy rate as high as 98% [38,39]; after analyzing the population variables of *Sander lucioperca* (Perciformes, Percidae), the comprehensive discrimination rate was 86.5%. In comparison, the external morphological differences between male and female perch are not significant enough, resulting in limited accuracy of discriminant analysis. This indicates that relying solely on these external morphological characteristics for sex discrimination has limitations in practical applications. To improve accuracy, molecular marker techniques or the excavation of more discriminative morphological indicators could be considered [40]. Based on the established growth equations for *P. fluviatilis*, a multi-dimensional analysis of its growth characteristics can be conducted. From the perspective of body length (L_t_) and body weight (W_t_) growth equations, both follow the typical form of the Logistic growth model. That is, as time t progresses, the growth gradually approaches the limit value under environmental carrying capacity (with the maximum body length being approximately 652.866 units and the maximum body weight approximately 4990.21 units). This is consistent with the growth pattern of fish restricted by resources in both natural and aquaculture environments, reflecting that the growth of *P. fluviatilis* has obvious asymptotic characteristics, which can be used as a basis for judging its growth stages (such as the rapid growth period of juveniles and the stable period of adults). The growth rate equations reveal that both body length and body weight growth rates show an exponential decay trend over time. In the body length growth rate equation, the exponential term dominates the decay process, meaning that the vitality of body length growth in *P. fluviatilis* gradually decreases with the development process, and the body length may expand more rapidly in the juvenile stage. The body weight growth rate equation shows that body weight growth is synergistically driven by body length growth in the early stage (increasing with the increase of t), while in the later stage, it is restricted by asymptotic characteristics, resulting in a decline in the growth rate. It presents a complex dynamic of “first rising and then falling” or continuous decay but regulated by synergistic terms, which is consistent with the characteristic that body weight growth in fish often lags behind body length and is affected by factors such as condition factor. The growth acceleration equations further deepen the understanding of growth changes. The body length acceleration is negative and decays exponentially over time, indicating that body length growth is always in a decelerating state, and the deceleration amplitude slows down over time. The deceleration of body length growth is more significant in the juvenile stage, and gradually stabilizes in the later stage [41,42].

As an important economic fish in the Irtysh River Basin [43], the age and growth characteristics of *P. fluviatilis* have always been key focuses in fishery resource research and management. Through in-depth analysis of perch samples, this study aims to reveal their age structure, growth patterns, and the complex relationships between these characteristics and the ecological environment.

In terms of age structure, the age distribution of *P. fluviatilis* in this study exhibits specific characteristics. Unlike the perch in Tangba Lake and Shuifeng [5,44] Reservoir studied by Wei Wenyan, Xu Haoran, and others, the age structure of perch in the Irtysh River Basin is relatively more low-aged. Perch captured in some river sections are predominantly 1-year-old, though a certain number of older individuals still exist. This difference may stem from the more complex and variable ecological environment of the Irtysh River compared to Sayram Lake, including differences in water flow, water temperature, and food resource distribution. As a transnational river, the habitat conditions of different sections of the Irtysh River vary significantly. For example, the Kalasuke Reservoir section in the Irtysh River Basin is closer to the north, with longer annual ice-covered periods, scarcer food resources, and increased human fishing pressure, which has led to a more pronounced low-aged structure in perch and gradually reduced survival opportunities for older individuals [14].

In terms of growth characteristics, *P. fluviatilis* exhibits a unique growth pattern. Fitting with the von Bertalanffy model reveals significant variations in growth rates across different age groups. Juvenile fish grow rapidly, similar to the growth patterns of many fish species, as this allows them to quickly reach a certain body size in the early stages to enhance survival competitiveness. Growth rates gradually slow down with age. Compared to *Triplophysa strauchii* [14], perch have a relatively earlier inflection age of growth but a longer rapid growth period. This may be related to the fishing pressure and relatively lower water temperatures in the Kalasuke Reservoir section of the Irtysh River Basin, where perch have a shorter time interval to obtain sufficient nutrition and less energy to support sustained growth. Growth is also influenced by sex. Due to the high nutritional requirements of ovarian development, female perch allocate more energy to gonadal development in the early growth stage, resulting in relatively slower growth rates and a later inflection age compared to males during certain phases. This is consistent with the phenomenon observed in species such as *Capoeta bilineata*, where the growth inflection point of females is later than that of males [45].

In terms of the relationship between growth and body weight, perch generally exhibit an allometric growth pattern dominated by body length increase (b < 3). An increase in body length may enable perch to expand their activity range, occupy new habitats or feeding grounds more quickly, reduce intraspecific competition, and enhance motor capabilities. Larger body length increases the relative area of the caudal fin, improving swimming speed and mobility to escape predators such as northern pike (*Esox lucius*) and pikeperch. Body length growth also serves as a prerequisite for sexual maturity: some studies indicate that female perch only become reproductively capable when reaching 15–20 cm in length. Through this growth pattern, perch have occupied a unique ecological niche in the Kalasuke Reservoir section of the Irtysh River Basin [46].

The condition factor of *P. fluviatilis* is also an important indicator reflecting its growth and nutritional status. Generally, the condition factor decreases with an increase in body length, which is consistent with previous research conclusions [47]. In this study, female perch captured in May had significantly lower condition factors than males because they had just completed the breeding season, and spawning led to a substantial consumption of their energy reserves. Meanwhile, males allocate most of their energy to maintenance and growth, accumulating relatively higher energy reserves before the breeding season to use for courtship competition and improve reproductive success. This indicates that energy allocation balance is crucial in the growth and reproduction of perch, and reasonable energy allocation strategies help perch better adapt to the environment and ensure the healthy continuation of the population [30].

## 5. Conclusions

This study is dedicated to the morphology, age structure, and growth of *P. fluviatilis* in the Kalasuke Reservoir section of the Irtysh River Basin. The *P. fluviatilis* has evolved a typical streamlined body shape, combining swimming efficiency and turning flexibility to adapt to the alternating rapids and slow-flowing habitats of the Irtysh River. The optimized development of its visual organs helps it accurately locate prey and avoid risks in turbid or low-light environments. Although sexual dimorphism in external morphology is not significant, female perch demonstrate a trade-off strategy between reproduction and growth through energy allocation adjustments, reflecting an adaptive mechanism for population continuation. The age structure of *P. fluviatilis* in the Kalasuke Reservoir section of the Irtysh River Basin exhibits a significant low-age trend, reflecting the combined effects of low water temperature, food limitation, and anthropogenic fishing pressure. The von Bertalanffy growth model indicates that rapid body length growth (b < 3) during the juvenile stage serves as a key strategy for expanding activity ranges and advancing sexual maturity. In contrast, female perch show a later growth inflection age than males due to the energetic demands of gonadal development, highlighting sex-specific differences in energy allocation. Ecological factors significantly influence growth dynamics: high-quality water and abundant food resources support basic growth requirements, but the introduction of alien species such as pikeperch (*Sander lucioperca*) has intensified resource competition. This has prompted some individuals to accelerate sexual maturity as a survival strategy to ensure population persistence. The pattern of condition factor decreasing with increasing body length, and the differences in sexual energy reserves during the breeding season, further confirm the dynamic adaptation of *P. fluviatilis* to environmental pressures during growth and reproduction. This study confirms that the morphological and growth characteristics of perch are evolutionary outcomes of long-term adaptation to the cold-water environment of the Irtysh River Basin, but its population is facing dual challenges of fishing pressure and ecological competition. Based on the low-age trend, the following measures are proposed: implement segmented fishing quotas by river section, restrict juvenile fish harvesting, and protect breeding opportunities for older individuals; monitor the continuous impact of alien species on *P. fluviatilis* habitats, and combine molecular marker techniques to identify sex-specific morphological indicators and improve sex discrimination accuracy; and develop integrated conservation strategies to maintain biodiversity and ecological stability in the Kalasuke Reservoir.

## Figures and Tables

**Table 1:** Morphometric traits of the *Perca fluviatilis* (*n* = 296, mm).

Traits	Range	Mean ± SD ^1^
Total length	119.87–355.10	153.45 ± 34.79
Body length	100.53–305.31	130.22 ± 29.63
Body width	10.36–56.02	18.47 ± 6.57
Body depth	19.74–90.13	33.71 ± 9.36
Head length	26.12–99.51	39.73 ± 10.07
Snout length	7.21–29.00	11.55 ± 3.27
Eye diameter	4.92–18.01	8.25 ± 1.68
Eye spacing	6.12–27.02	10.32 ± 3.19
Caudal peduncle length	14.58–76.53	24.66 ± 9.07
Caudal peduncle height	5.31–24.12	9.86 ± 2.50
Mouth cleft wide	5.35–24.05	9.21 ± 2.77
Mouth cleft high	4.60–20.07	10.01 ± 3.01

Note: SD ^1^ represents standard deviation.

**Table 2:** Ratio of measurable characteristics of *Perca fluviatilis*. (*n* = 296; mm).

Indicator	Female		Male	
	Range	Mean ± SD	Range	Mean ± SD ^1^
Total length/body length	1.02–1.26	1.18 ± 0.04	1.06–1.57	1.18 ± 0.05
Body length/body width	4.75–10.57	7.33 ± 1.31	4.41–12.27	7.29 ± 1.25
Body length/body depth	2.79–5.74	3.89 ± 0.35	3.08–4.78	3.91 ± 0.27
Body depth/body width	1.24–2.96	1.89 ± 0.31	1.31–3.10	1.86 ± 0.29
Body length/head length	2.71–3.87	3.29 ± 0.20	2.78–4.28	3.30 ± 0.19
Body length/caudal peduncle length	3.91–7.83	5.32 ± 0.96	3.88–8.98	5.59 ± 1.02
Body length/eye diameter	10.74–22.54	16.12 ± 1.81	10.74–23.45	15.71 ± 1.87
Body length/eye spacing	7.80–16.81	12.80 ± 1.97	8.27–19.08	13.02 ± 1.71
Head length/snout length	2.76–4.73	3.48 ± 0.38	2.21–4.88	3.50 ± 0.47
Head length/eye diameter	3.16–7.04	4.92 ± 0.60	2.90–6.92	4.77 ± 0.56
Head length/caudal peduncle length	1.17–2.42	1.62 ± 0.30	1.06–2.52	1.71 ± 0.32
Head length/mouth cleft wide	2.85–6.54	4.39 ± 0.73	3.06–7.51	4.43 ± 0.70
Head length/eye spacing	2.25–4.95	3.90 ± 0.60	2.43–5.93	3.95 ± 0.55
Caudal peduncle length/caudal peduncle height	1.79–3.43	2.55 ± 0.40	1.28–3.64	2.47 ± 0.43

Note: ^1^ SD represents standard deviation.

**Table 3:** *Perca fluviatilis* measurable trait ratios’ characteristic values in PC1–PC3. (*n* = 296; mm).

Character	PC1	PC2	PC3
Total length/body length	0.13416	0.13796	−0.04618
Body length/body width	0.41879	0.08958	0.30886
Body length/body depth	0.13946	−0.1017	0.33047
Body depth/body width	0.39542	0.15036	0.19541
Body length/head length	−0.00747	−0.13646	0.44913
Body length/caudal peduncle length	0.4215	−0.37641	−0.05525
Body length/eye diameter	0.1431	0.0292	−0.36795
Body length/eye spacing	0.28035	0.40419	0.14773
Head length/snout length	0.05	0.1998	−0.02699
Head length/eye diameter	0.12882	0.09162	−0.56315
Head length/caudal peduncle length	0.4117	−0.32276	−0.18771
Head length/mouth cleft wide	0.13714	0.33371	−0.17245
Head length/eye spacing	0.26961	0.42866	−0.02699
Caudal peduncle length/caudal peduncle height	−0.2719	0.40971	0.09757

**Table 4:** Truss structure distances of *Perca fluviatilis* (*n* = 296, mm).

Location	Range	Mean ± SD ^1^
A-B	5.96–26.60	16.86 ± 3.48
B-C	9.34–50.57	30.62 ± 6.98
C-D	3.54–17.48	11.71 ± 2.67
D-E	3.44–60.54	38.63 ± 8.79
E-F	2.35–9.15	4.87 ± 1.21
F-G	4.73–50.41	30.23 ± 7.75
G-H	6.07–53.81	17.68 ± 4.76
H-I	7.17–45.64	27.70 ± 6.23
A-I	8.32–34.28	19.44 ± 3.86
A-H	10.97–69.72	46.63 ± 9.66
A-C	15.64–69.80	46.27 ± 9.51
B-H	7.69–51.25	32.88 ± 7.28
B-I	3.68–24.28	11.21 ± 2.33
C-I	8.12–52.57	29.22 ± 6.61
C-H	3.45–20.26	12.26 ± 2.84
C-G	7.44–48.77	21.09 ± 4.71
C-F	16.93–77.53	50.49 ± 10.96
D-H	5.15–38.59	14.28 ± 3.90
D-G	3.89–36.53	11.58 ± 3.25
D-F	12.13–60.37	38.61 ± 8.65
E-G	8.61–51.32	31.49 ± 7.54

Note: ^1^ A. Tip of the snout; B. back of the occiput; C. origin of the dorsal fin; D. end of the dorsal fin base; E. origin of the dorsal part of the caudal fin; F. origin of the ventral part of the caudal fin; G. end of the anal fin base; H. base of the pelvic fin; I. origin of pectoral fin.

## Data Availability

The datasets presented in this article are not readily available because the data are part of an ongoing study or due to technical/time limitations. Requests to access the datasets should be directed to Jie Wei.
